# Infiltrative growth pattern of prostate cancer is associated with lower uptake on PSMA PET and reduced diffusion restriction on mpMRI

**DOI:** 10.1007/s00259-022-05787-9

**Published:** 2022-04-18

**Authors:** Riccardo Laudicella, Jan H. Rüschoff, Daniela A. Ferraro, Muriel D. Brada, Daniel Hausmann, Iliana Mebert, Alexander Maurer, Thomas Hermanns, Daniel Eberli, Niels J. Rupp, Irene A. Burger

**Affiliations:** 1 Department of Nuclear Medicine, University Hospital Zurich, University of Zurich, Rämistrasse 10, 8091 Zurich, Switzerland; 2 Department of Biomedical and Dental Sciences and Morpho-Functional Imaging, Nuclear Medicine Unit, University of Messina, Messina, Italy; 3 Department of Pathology and Molecular Pathology, University Hospital Zurich, University of Zurich, Zurich, Switzerland; 4 Department of Radiology and Oncology, Faculdade de Medicina, FMUSP, Universidade de Sao Paulo, Sao Paulo, Brazil; 5 Department of Radiology, Kantonsspital Baden, Baden, Switzerland; 6 Department of Clinical Radiology and Nuclear Medicine, Medical Faculty Mannheim, University Medical Center Mannheim, Heidelberg University, Mannheim, Germany; 7 Department of Urology, University Hospital Zurich, University of Zurich, Zurich, Switzerland; 8 Department of Nuclear Medicine, Kantonsspital Baden, Baden, Switzerland

**Keywords:** Diffusion-weighted imaging, MRI, Prostate cancer, PSMA PET/MRI, Radical prostatectomy

## Abstract

**Purpose:**

Recently, a significant association was shown between novel growth patterns on histopathology of prostate cancer (PCa) and prostate-specific membrane antigen (PSMA) uptake on [^68^Ga]PSMA-PET. It is the aim of this study to evaluate the association between these growth patterns and ADC (mm^2^/1000 s) values in comparison to [^68^Ga]PSMA uptake on PET/MRI.

**Methods:**

We retrospectively evaluated patients who underwent [^68^Ga]PSMA PET/MRI for staging or biopsy guidance, followed by radical prostatectomy at our institution between 07/2016 and 01/2020. The dominant lesion per patient was selected based on histopathology and correlated to PET/MRI in a multidisciplinary meeting, and quantified using SUV_max_ for PSMA uptake and ADC_mean_ for diffusion restriction. PCa growth pattern was classified as expansive (EXP) or infiltrative (INF) according to its properties of forming a tumoral mass or infiltrating diffusely between benign glands by two independent pathologists. Furthermore, the corresponding WHO2016 ISUP tumor grade was evaluated. The *t* test was used to compare means, Pearson’s test for categorical correlation, Cohen’s kappa test for interrater agreement, and ROC curve to determine the best cutoff.

**Results:**

Sixty-two patients were included (mean PSA 11.7 ± 12.5). The interrater agreement between both pathologists was almost perfect with *κ* = 0.81. While 25 lesions had an EXP-growth with an ADC_mean_ of 0.777 ± 0.109, 37 showed an INF-growth with a significantly higher ADC_mean_ of 1.079 ± 0.262 (*p* < 0.001). We also observed a significant difference regarding PSMA SUV_max_ for the EXP-growth (19.2 ± 10.9) versus the INF-growth (9.4 ± 6.2, *p* < 0.001). Within the lesions encompassing the EXP- or the INF-growth, no significant correlation between the ISUP groups and ADC_mean_ could be observed (*p* = 0.982 and *p* = 0.861, respectively).

**Conclusion:**

PCa with INF-growth showed significantly lower SUV_max_ and higher ADC_mean_ values compared to PCa with EXP-growth. Within the growth groups, ADC_mean_ values were independent from ISUP grading.

**Supplementary information:**

The online version contains supplementary material available at 10.1007/s00259-022-05787-9.

## Introduction

Prostate cancer (PCa) represents the second global cause of death from cancer, with an incidence of 190,000 new patients and over 33,000 related deaths per year in the USA [1]. In many institutions, patients with a suspected PCa due to an increased prostate-specific antigen (PSA) value or altered digital-rectal exam results will undergo an ultrasound (US)-guided biopsy procedure. However, US-guided biopsy can miss significant cancer in up to 25% of patients [2]; therefore, the recent joint EAU-EANM-ESTRO-ESUR-SIOG guideline suggests performing multiparametric magnetic resonance imaging (mpMRI)-guided biopsy or MRI-guided US-fusion biopsy [3, 4]. The introduction of mpMRI for PCa detection has induced significant improvement in the early detection of PCa. The mpMRI accuracy was further improved, and inter-reader variability was reduced, thanks to the PI-RADS score system. PIRADS 2.1 criteria are based on a 5-point scale using a combination of mpMRI findings (T2, diffusion-weighted imaging—DWI—with dynamic contrast-enhanced—DCE), to predict the presence of a clinically significant prostate cancer (csPCa). The definition of csPCa on biopsy cores is based on histopathology with a Gleason score (GS) ≥ 4 + 3 (grade 7b and higher) or a maximum cancer core length ≥ 6 mm according to the Epstein criteria [5]. T2-weighted images are mostly used to evaluate prostate gland anatomy and to identify suspicious lesions with a concurrent morphological characterization. DWI represents a core sequence used for PCa able to evaluate the cell density exploiting the diffusion of water molecules due to their thermal energy. A positive correlation between GS and the reduction in apparent diffusion coefficient (ADC) has been displayed in several studies enabling improved csPCa detection [6, 7]. Therefore, it has been shown that performing a mpMRI before a biopsy could avoid ≃30% of unnecessary biopsies and improve the csPCa detection by almost 15% [8, 9]. However, despite the adjusted PIRADS 2.1 version aimed to simplify the interpretation, mpMRI inter-reader reproducibility remains sub-optimal with reported kappa values between 0.37 and 0.48 [10]; false-positive results are also described in almost 20% of the patients [11, 12], and some aggressive tumors are negative on ADC maps [13, 14].

Recently, attention has focused on the new prostate-specific membrane antigen (PSMA) targeting PET tracer for PCa assessment [15]. It is well established that the PSMA uptake of the primary tumor is related to a higher GS and a worse prognosis [16]. However, preliminary results also showed significant PCa without PSMA uptake [17, 18]. Further histopathological analysis of PSMA-negative PCa showed that these tumors have a predominantly infiltrative (INF) rather than expansive (EXP) growth pattern [19]. This observation induces the hypothesis that growth patterns might also affect mpMRI diffusion sequences within cancer tissue and could cause false-negative results. Searching the literature, indeed a few publications mentioning a correlation between morphological patterns and ADC values were present [20–23]. However, there was no incorporation of tumor aggressiveness and growth pattern in correlation to ADC values; there is still no consensus on how sparse or intermixed benign glands are defined or how reliable such a definition is.

Therefore, we aimed to test the interrater agreement for the new growth patterns on histopathology and to correlate them with ADC values with respect to PSMA uptake and ISUP grades.

## Materials and methods

### Patients

**Fig. 1:**
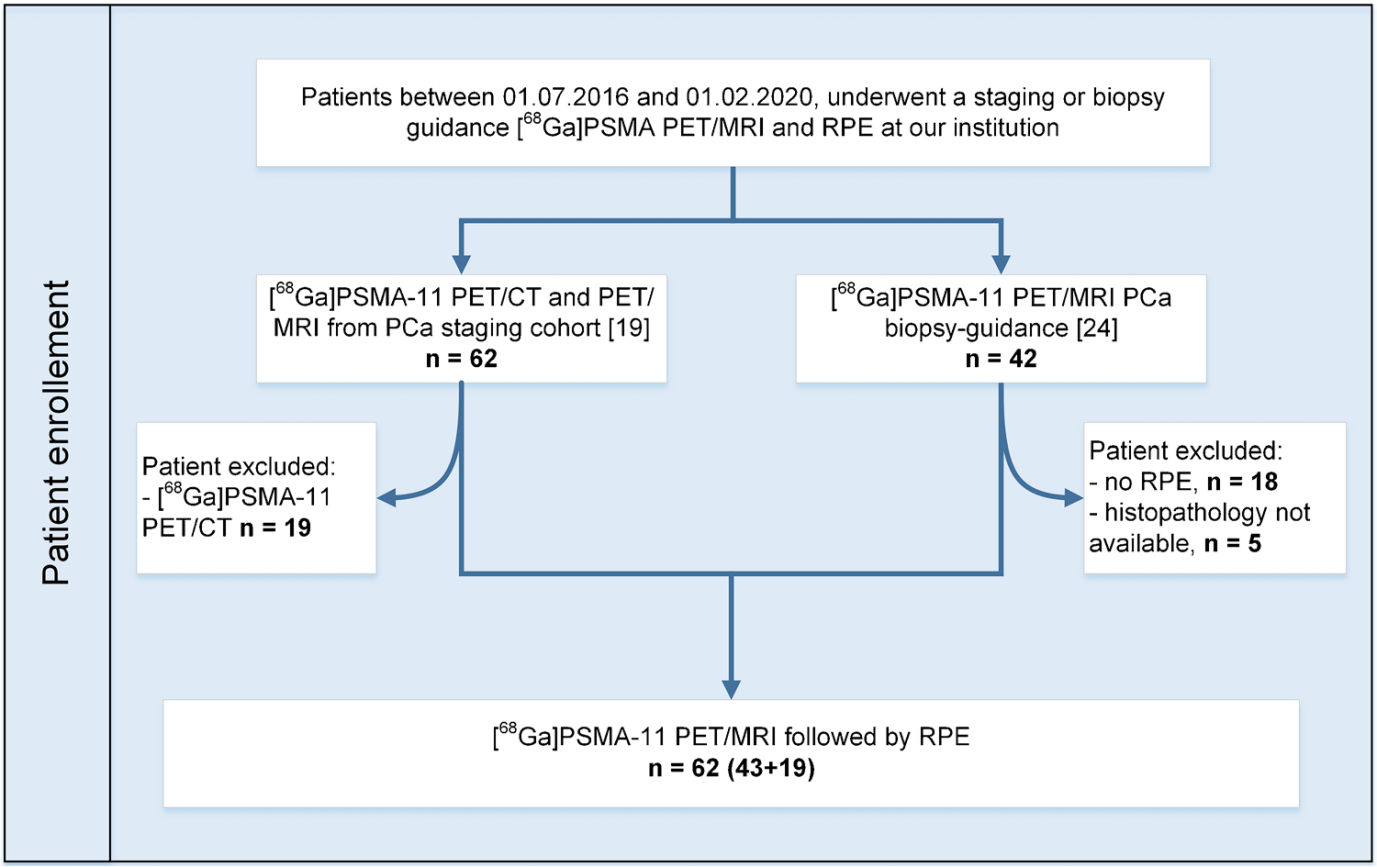
Patients’ selection and inclusion in the study

In this retrospective analysis, we included all PCa patients who, between 01/07/2016 and 01/02/2020, consecutively underwent a staging or prospectively a biopsy guidance [^68^Ga]PSMA PET/MRI. The study cohort comprises two groups (Fig. 1**)**. Based on the published analysis of staging [^68^Ga]PSMA-11 PET/MRI and PET/CT in correlation with histopathology [19], we included all patients that underwent PET/MRI (*n* = 43), group 1. This cohort was limited to mostly higher risk tumors; therefore, we added all patients that underwent [^68^Ga]PSMA-11 PET/MRI for biopsy guidance with RPE and histopathology samples available (*n* = 19), group 2 [24], aiming to improve the spectrum of the study and reduce the bias for high-risk disease only. Scans were performed before radical prostatectomy (RPE), executed at our institution using a robot-assisted transperitoneal laparoscopic approach with bilateral extended lymph nodal dissection (four-arm Da Vinci S system, Intuitive Surgical, Inc., USA). The study was approved by the institutional review board; all patients from group 1 signed a written general informed consent, and patients in group 2 signed the specific written informed consent of the prospective biopsy guidance study.

#### PET/MRI

All patients underwent [^68^Ga]PSMA-11 PET/MRI scans (SIGNA PET/3 T MRI, GE Healthcare, Waukesha, WI, USA) for one of the following indications: group (1) staging PCa (43/62 patients) with an administered [^68^Ga]PSMA-11 dose of 2 MBq/kg (mean activity 129.8 ± 21.1 MBq, range 81–160); and group (2) biopsy guidance (19/62 patients) with an administered [^68^Ga]PSMA-11 dose of 85 MBq per patients. Images were acquired 60 min after the injection of [^68^Ga]PSMA-11, starting with a whole-body MRI localizer scan. Subsequently, a 3D dual-echo, spoiled gradient recalled echo sequence (LAVA-FLEX) for attenuation correction, and a PET emission scan were acquired. The default number of bed positions was six, and the acquisition time per bed position was 2 min. The whole-body protocol also included dedicated sequences covering the pelvis, including a high-resolution T1-weighted LAVA-FLEX sequence, a T2-weighted fast recovery fast spin-echo sequence in two planes, and DWI (*b* values: 0, 300, and 1000). Details of MRI sequences are given in supplemental Table 1. PET acquisition for the whole-body protocol was in a 3D time of flight (TOF) mode, six-bed positions with 2-min acquisition time per bed position (axial FOV of 25 cm and overlap of 24%, matrix 256 × 256, 2 iterations, 28 subsets, with the sharpIR algorithm—GE Healthcare—and 5-mm filter cutoff). To reduce the radiopharmaceutical activity in the urinary system, furosemide was injected intravenously 30 min before the tracer injection (0.13 mg/kg), and the patients were asked to void before the scan. The institutional protocol followed the joint EANM-SNMMI procedure guidelines [25].

**Table 1:** Patients’ characteristics

	Total	INF growth	EXP growth	***p*** values
Number of patients	62	37	25	
Age (years)	64.2 ± 6.2 (51–78)	63.5 ± 5.8 (51–74)	65.0 ± 6.6 (52–78)	0.747
PSA before PET/MRI (ng/ml)	11.7 ± 12.5 (1.2–77.9)	9.1 ± 7.9 (1.2–44)	15.5 ± 16.4 (1.3–77.9)	0.069
ISUP biopsy grade ***n*** (%)				0.756
1	8/62 (13%)	6/37 (16%)	2/25 (8%)	
2	12/62 (19%)	8/37 (22%)	4/25 (16%)	
3	15/62 (24%)	9/37 (24%)	6/25 (24%)	
4	19/62 (31%)	10/37 (27%)	9/25 (36%)	
5	8/62 (13%)	4/37 (11%)	4/25 (16%)	
Time between PET/MRI and RPE				0.010
Days	72.9 ± 78 (1–481)	89.9 ± 93.7 (1–481)	47.6 ± 31.9 (1–116)	
ISUP RPE grade ***n*** (%)				0.050
1	0/62 (0%)	0/37 (0%)	0/25 (0%)	
2	14/62 (22%)	12/37 (32%)	2/25 (8%)	
3	27/62 (44%)	17/37 (46%)	10/25 (40%)	
4	13/62 (21%)	7/37 (19%)	6/25 (24%)	
5	8/62 (13%)	1/37 (3%)	7/25 (28%)	
Histopathology pattern				0.001
Acinar	43/62 (69%)	34/37 (91%)	9/25 (36%)	
Acinar + ductal	3/62 (5%)	2/37 (5%)	1/25 (4%)	
Acinar + intraductal	4/62 (6%)	2/37 (5%)	2/25 (8%)	
Acinar + cribriform	7/62 (11%)	0/37 (0%)	7/25 (28%)	
Acinar + ductal + cribriform	1/62 (1.6%)	0/37 (0%)	1/25 (4%)	
Acinar + intraductal + cribriform	3/62 (5%)	0/37 (0%)	3/25 (12%)	
Acinar + ductal + intraductal + cribriform	1/62 (1.6%)	0/37 (0%)	1/25 (4%)	

*INF*, infiltrative; *EXP*, expansive; *SD*, standard deviation; *PET/MRI*, positron emission tomography/magnetic resonance imaging; *PSA*, prostate-specific antigen; *ISUP*, International Society Of Urological Pathology; *RPE*, radical prostatectomy; *p*, statistical difference between the INF and EXP done with *t* test for continuous data and for ISUP grade and growth-pattern group according to Mann–Whitney test; in bold all the significant *p*-values

### Histopathology assessment

**Fig. 2:**
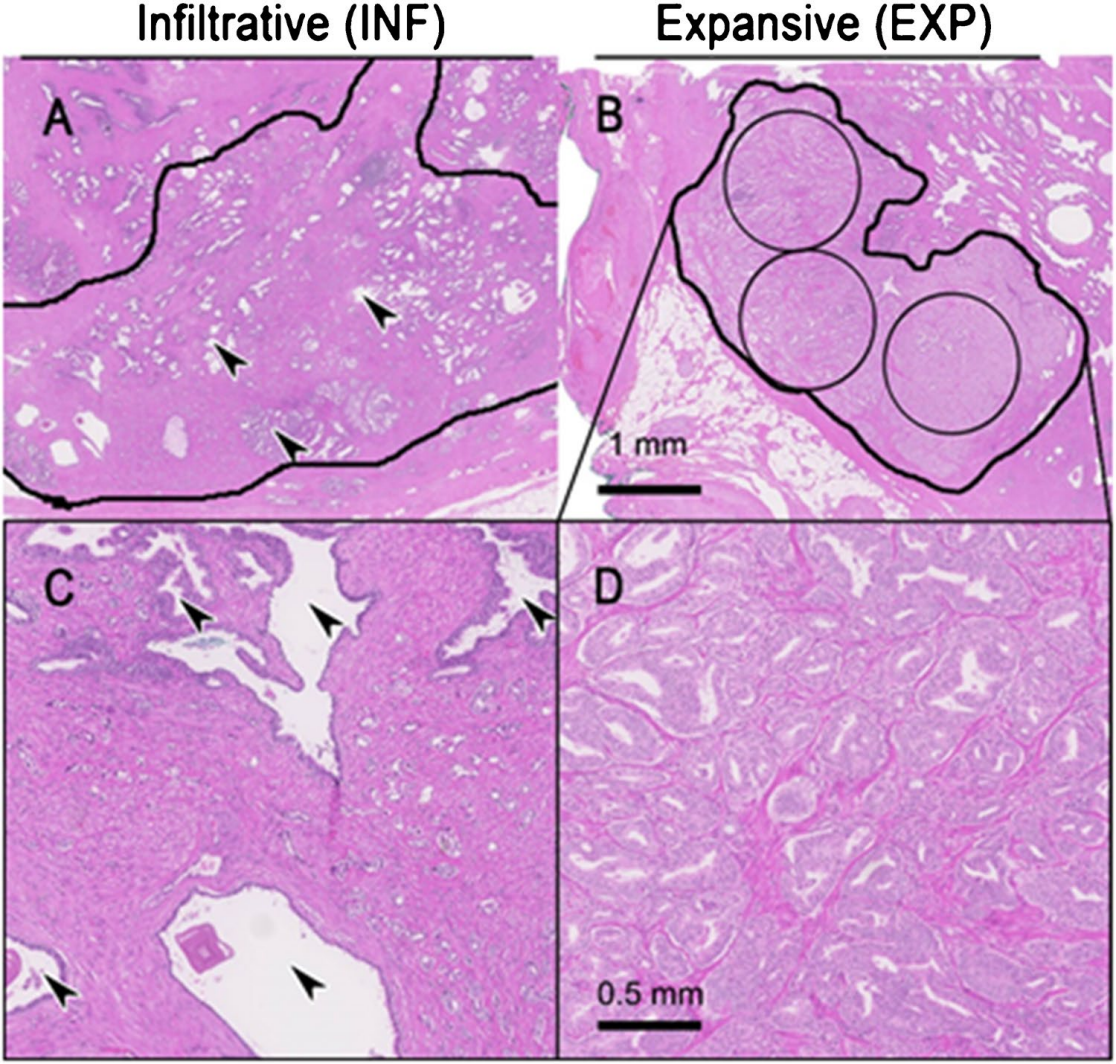
Examples of different growth patterns. **A**, **C** Example of infiltrative growth pattern (INF) defined by prostate carcinoma (circled) growing between benign glands (arrowheads). **B**, **D** Expansive growth pattern (EXP), which homogenously comprises tumor glands containing at least 3 circles of 5 mm^2^ (radius 1.26 mm). **A**, **B** Scale bar 1 mm. **C**, **D** Scale bar 0.5 mm

The histopathological 2-µm hematoxylin and eosin (H&E) slides corresponding to the investigated areas on mpMRI and PET scans were subjected to established WHO2016/International Society of Urological Pathology (ISUP) prognostic grade group grading [26] by two experienced genito-urinary pathologists (JHR, NJR). The pathologist selected the largest prostate cancer lesion per patient for further analysis to ensure accurate correlation with imaging data later on and reduce the risk of partial volume effects on imaging data. For each lesion, the tumor type, including acinar, ductal, intraductal, and cribriform differentiation, was recorded. Notably, a ductal pattern was assigned to tumors showing true papillary formation according to an international inter-observer study [27]. Intraductal and cribriform differentiation was defined based on a recently published study [28]. Also, two different growth patterns were evaluated: (I) The EXP growth was defined for tumors, which showed a histological tumoral mass comprising at least 3 × 5 mm^2^ (radius 1.26 mm) sized circles containing solely and dense tumoral tissue without intermingled benign glands. (II) If these criteria were not fulfilled and tumors were lacking, a tumoral mass with the previously mentioned criteria, and infiltration between benign glands was noticeable, an INF growth was documented. The INF growth is characterized by a typical irregular invasion front and evident tumor cell infiltration between benign glands. Alternatively, an INF growth is present when the tumor invaded diffusively with a widespread penetration into the normal tissue. Differently, an EXP growth is characterized by a smooth and broad invasion front with minimal/absent tumor cells dissociation (Fig. 2). The tumor maximum diameter on histopathology was measured for all those tumors’ lesions fixed in formalin.

### Image analysis

**Fig. 3:**
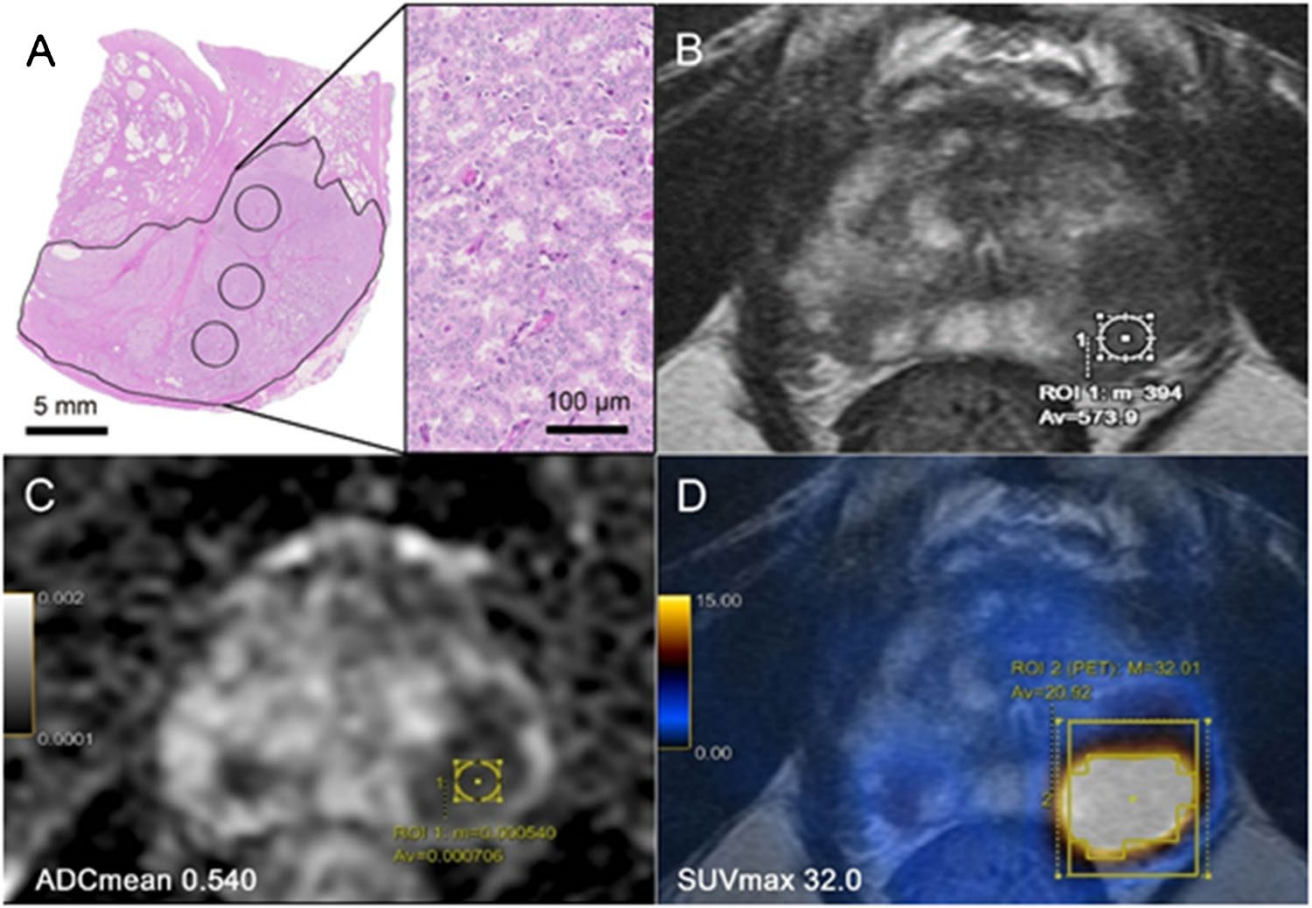
Region of interest (ROI) selection. Example of a 74-year-old patient with PSA of 20 ng/ml. Subsequently, he underwent radical prostatectomy (GS = 4 + 4, ISUP 4). **A** Histopathology slide showing an expansive growth pattern of the “dominant lesion’’ on the left posterior part of the prostatic peripheral zone. **B** Corresponding axial T2-weighted MRI showing a large hypointense area. **C** Axial ADC MRI with low ADC_mean_ values = 0.540. **D** axial [^68^Ga]PSMA PET/MRI with high PSMA-uptake (SUV_max_ of 32.0)

According to the histopathology, the selected “dominant lesion” per patient on the hematoxylin and eosin slides was compared to the PET/MRI images in a multidisciplinary meeting (including pathology, radiology, and nuclear medicine). A region of interest (ROI) was inserted over the area selected on pathology on both PSMA images and ADC maps (mm^2^/1000 s) (Fig. 3). Semi-quantitative image parameters were obtained for both modalities, including the average and minimum ADC values (ADC_mean_ and ADC_min_, mm^2^/1000 s), and the corresponding [^68^Ga]PSMA-11 uptake as maximum and average standard uptake values (SUV_max_ and SUV_mean_). Image analysis was done in consensus for PSMA PET/MRI by IAB (radiologist and nuclear medicine physician, with 10 years of experience) and mpMRI analysis by DH (radiologist, with 12 years of experience). All the PET/MRI images were analyzed in a dedicated review workstation (Advantage Workstation, Version 4.7, GE Healthcare), which enables the review of the PET and MRI images side by side and in fused mode. All mpMRI images were double-checked for bleeding artefacts in the region of interest on T1-weighted images; no case had to be excluded.

### Statistical analysis

Statistical analyses were performed using SPSS statistics software, version 26 (IBM). Descriptive analyses were used to display patient data as mean and range. To evaluate the interrater agreement between pathologists, Cohen’s Kappa test was used, with *–* < 0 as poor, (0.01–0.2) slight, (0.21–0.40) fair, (0.41–0.6) moderate, (0.61–0.80) substantial, and (0.81–0.91) almost perfect agreement. The *t* test was used to compare the mean values. The correlation between histopathological growth pattern (INF or EXP) and lesions’ ISUP grading was assessed with the Pearson’s test; the correlation between lesions’ ISUP grading and semiquantitative parameters was assessed with the Spearman’s test. A *p* value of less than 0.05 was considered statistically significant, and for multiple testing, the *p* value was adjusted according to Bonferroni. The ability of PET and MRI parameters to predict growth patterns was assessed with receiver operating characteristics (ROC) analysis. Scatter plots for PET and MRI values regarding maximum tumor diameter on histopathology and ISUP grade were given to show the interaction between size, ISUP grade, and imaging parameters. Statistical analyses were performed by IAB and RL (nuclear medicine physician).

## Results

Sixty-two (62) patients (mean age 64.2 ± 6.2 years; mean PSA 11.7 ± 12.5 ng/ml) underwent [^68^Ga]PSMA-11 PET/MRI, a median of 50.5 days (72.9 ± 78, range 1–481 days) before RPE. Forty-three patients underwent a PSMA PET/MRI for staging and 19 patients for biopsy guidance. The dominant lesion had an EXP growth in 25 patients (40.3%), while 37 lesions had an INF growth (59.7%). The interrater agreement between both pathologists was almost perfect with *κ* = 0.81. For further analysis, the results of the more experienced reader were selected.

**Fig. 4:**
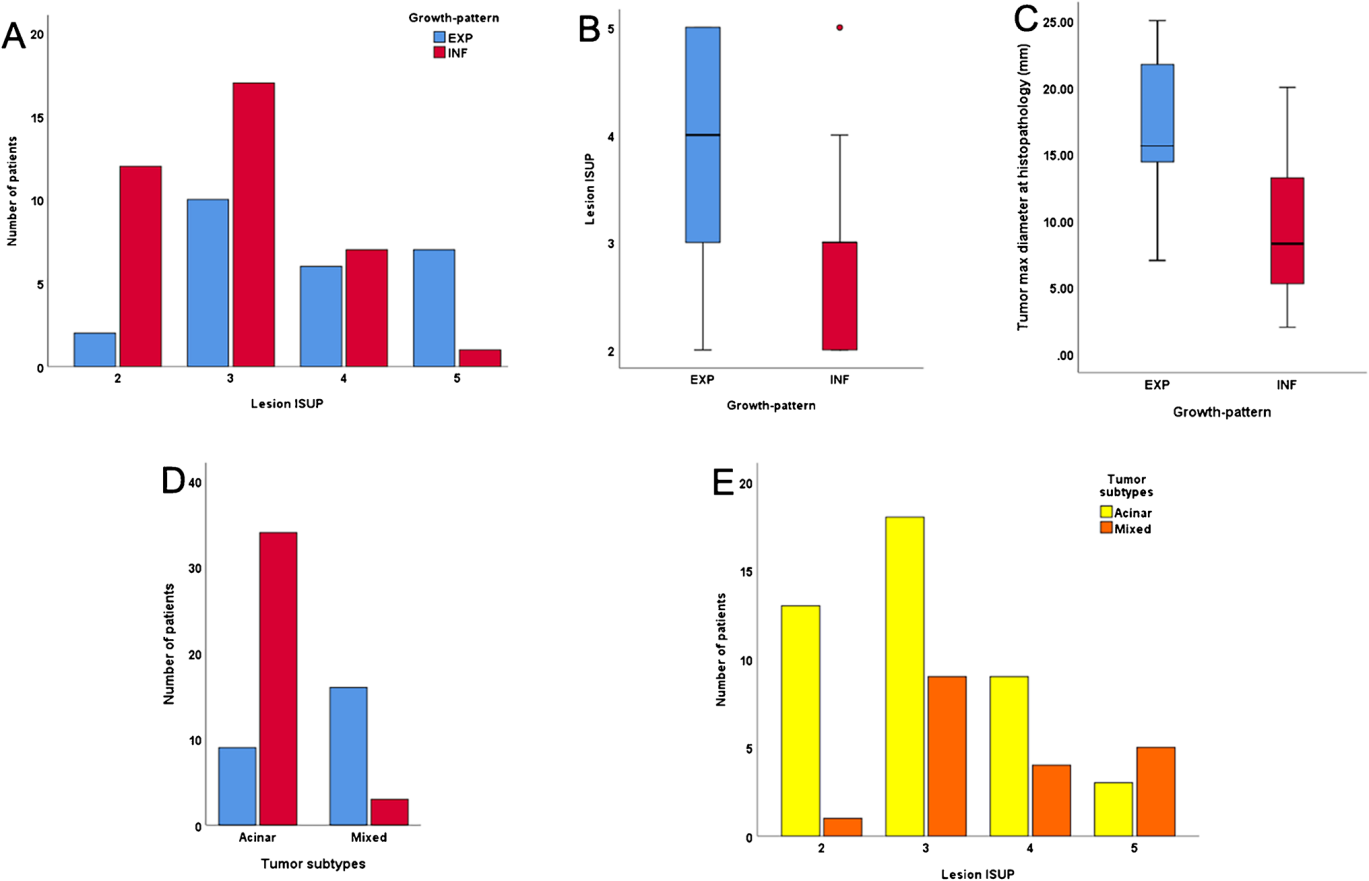
**A** Bar graphs for patients’ distribution according to RPE ISUP grade and growth pattern and box plot illustrations. **B** RPE ISUP distribution and **C** the maximum tumor diameter on histopathology according to growth-pattern. **D** Illustration of the distribution of PCa subtypes divided into either acinar or mixed (including combinations of acinar, ductal, intraductal and/or cribriform) according to growth pattern and **E** ISUP on histopathology

Lesions with INF growth had a lower ISUP grade compared to lesions with EXP growth (Fig. 4A). On RPE, the ISUP categories were significantly lower for INF growth compared to EXP growth (*p* = 0.001), with a median ISUP of three for INF growth tumors (mean ISUP = 2.9 ± 0.78), versus a median ISUP of four for EXP growth (mean ISUP = 3.7 ± 0.96) (Fig. 4B). Size on histopathology was measurable in 60 of the 62 lesions (in two patients, the dominant tumor was in the fresh frozen section of the apex). Among the measurable 60 lesions, the INF growth tumors were significantly smaller than the EXP growth (*p* < 0.001) with a mean tumor max diameter of 9.1 ± 4.8 mm and 17.2 ± 4.5 mm, respectively (Fig. 4C). Patients’ main characteristics are described in Table 1.

### Correlation between tumor subtypes and growth pattern

Among the whole cohort, we also analyzed the histopathology tumors subtypes. Overall, there were 43/62 acinar and 19/62 mixed subtypes described as acinar with an additional ductal, intraductal, or cribriform component. Significantly, more tumors with pure acinar characteristics had INF growth, while mixed tumor types tended to be EXP (Fig. 4D). Furthermore, mixed tumors had overall higher ISUP grades than acinar carcinoma (Fig. 4E). Of the 25 lesions with EXP growth, 12 lesions showed cribriform characteristics, six had an intraductal component, and five showed more than two morphology patterns (e.g., acinar, intraductal, and cribriform). Only 3 of the 37 in the infiltrative group were mixed with two lesions having acinar and intraductal components and one lesion with acinar and ductal components (Table 1).

### Correlation between growth pattern and imaging parameters

#### Overall distribution

The mean PSMA SUV_max_, SUV_mean_, ADC_mean_, and ADC_min_ values of the whole cohort were 13.2 ± 9.7, 7.4 ± 4.9, 0.957 ± 0.260, and 0.708 ± 0.217, respectively. Semi-quantitative values of the presented cohort are described in Table 2. We observed significantly higher uptake on PSMA PET in lesions with EXP growth (SUV_max_ 19.2 ± 10.9/SUVmean 9.6 ± 5.6) versus INF-growth (SUV_max_ 9.3 ± 6.2, *p* < 0.001/SUV_mean_ 6.0 ± 3.7, *p* = 0.01), as shown in Supplemental Fig. 1A-B. In analogy, we observed significantly more diffusion restriction in lesions with EXP growth (ADC_mean_ 0.777 ± 0.109/ADC_min_ 0.576 ± 0.110) versus INF growth (ADC_mean_ 1.079 ± 0.262, *p* < 0.001/ADC_min_ 0.797 ± 0.226, *p* < 0.001), Supplemental Fig. 1C-D.

**Table 2:** Semi-quantitative values for DWI and PSMA uptake

Semi-quantitative parameters	Total	INF growth	EXP growth	*p* values
SUV_max_ (mean/SD)	13.2 ± 9.7	9.3 ± 6.2	19.2 ± 10.9	** < 0.001**
SUV_mean_ (mean/SD)	7.5 ± 4.9	6.0 ± 3.7	9.6 ± 5.6	**0.01**
ADC_min_ (mean/SD)	0.708 ± 0.217	0.797 ± 0.226	0.576 ± 0.110	** < 0.001**
ADC_mean_ (mean/SD)	0.957 ± 0.260	1.079 ± 0.262	0.777 ± 0.109	** < 0.001**

*INF*, infiltrative; *EXP*, expansive; *SUV*, standardized uptake value; *max*, maximum; *min*, minimum; *SD*, standard deviation; *ADC*, apparent diffusion coefficient; in bold all the significant *p*-values

### Correlation between imaging parameters and ISUP

**Fig. 5:**
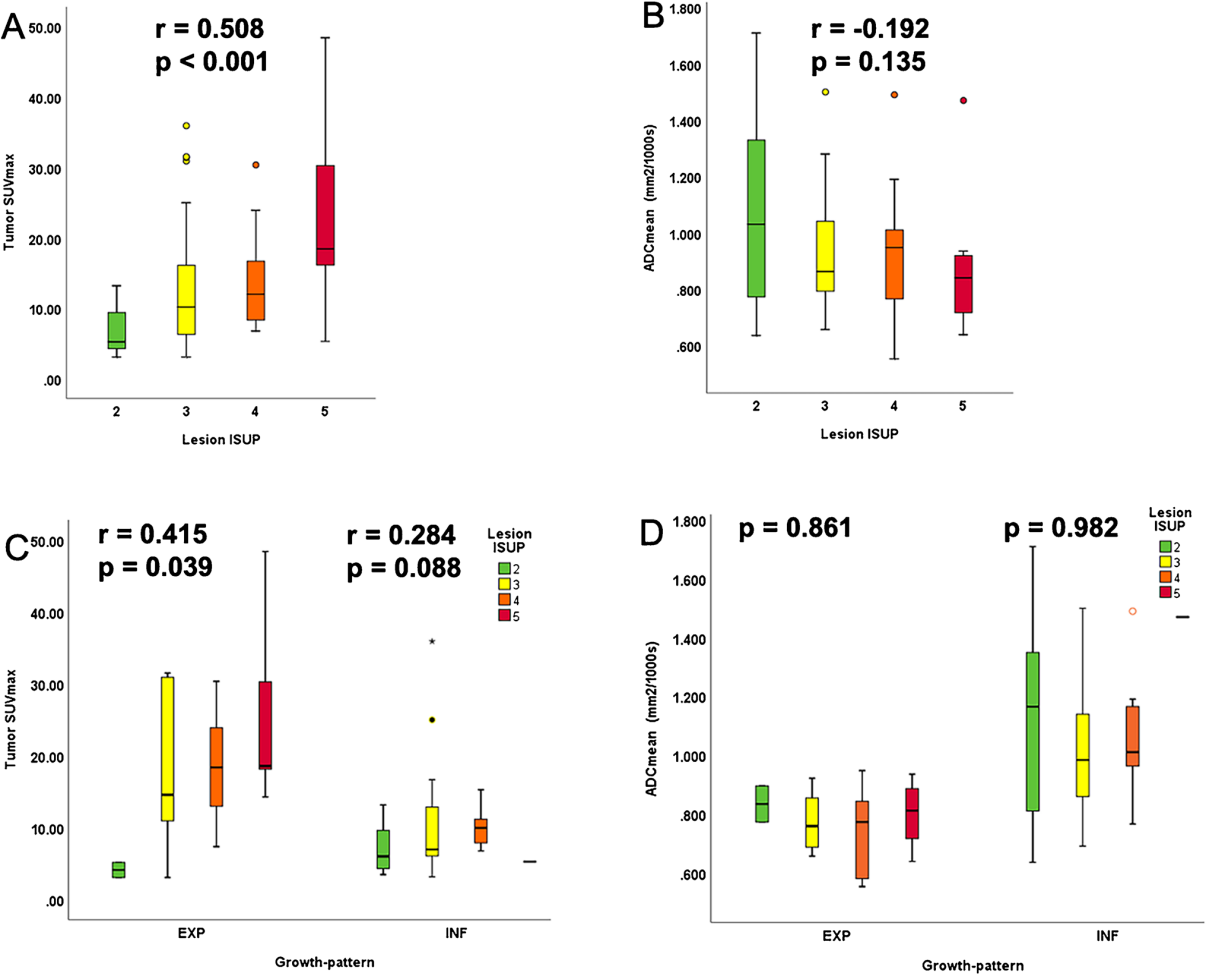
Box plots illustrating the relationship between imaging parameters and RPE ISUP grade. **A** PSMA SUV_max_ had a positive correlation to ISUP (*r* = 0.508, *p* < 0.001). **B** ADC_mean_ a negative trend in correlation to ISUP (*r* =  − 0.192, *p* = 0.135) for all lesions. **C** A significant positive correlation for SUV_max_ with ISUP was confirmed for EXP growth (*r* = 0.415, *p* = 0.039) but not for INF growth (*r* = 0.284, *p* = 0.088). **D** For subdivided growth patterns, the ADC_mean_ had no correlation to ISUP (*p* = 0.861 for EXP, and *p* = 0.982 for INF)

Overall, as shown in Table 3, we confirmed the significant positive correlation between lesions’ PSMA SUV_max_ and ISUP grade (*r* = 0.508, *p* < 0.001) (Fig. 5A) alongside trend for a negative correlation between ADC_mean_ and ISUP (*r* =  − 0.192, *p* = 0.135) (Fig. 5B). For PSMA uptake, the SUV_max_ still correlated significantly with ISUP for lesions with EXP growth (*r* = 0.415, *p* = 0.039) (Fig. 5C). However, if the analysis was performed for ADC_mean_ in both growth patterns separately, there was no significant correlation between ADC_mean_ and ISUP (EXP growth: *r* = 0.037, p = 0.861; INF growth: *r* =  − 0.004, *p* = 0.982; Fig. 5D).

**Table 3:** Spearman’s correlation between semi-quantitative values and lesions’ ISUP grading

Semi-quantitative parameters	Whole cohort (*n* = 62)	INF growth *n* = 37	EXP growth *n* = 25
SUV_max_	**0.508 (*p* < 0.001)**	0.284 (*p* = 0.088)	**0.415 (*p* = 0.039)**
SUV_mean_	**0.453 (*p* < 0.001)**	0.267 (*p* = 0.110)	0.343 (*p* = 0.94)
ADC_mean_	− 0.192 (*p* = 0.135)	− 0.004 (*p* = 0.982)	0.037 (*p* = 0.861)
ADC_min_	− 0.104 (*p* = 0.423)	0.024 (*p* = 0.886)	0.144 (*p* = 0.491)

*INF*, infiltrative; *EXP*, expansive; *SUV*, standardized uptake value; *max*, maximum; *ADC*, apparent diffusion coefficient; *ISUP*, WHO 2016 Gleason score prognostic grade group; in bold all the significant *p*-values

### Prediction of growth pattern based on imaging parameters

**Fig. 6:**
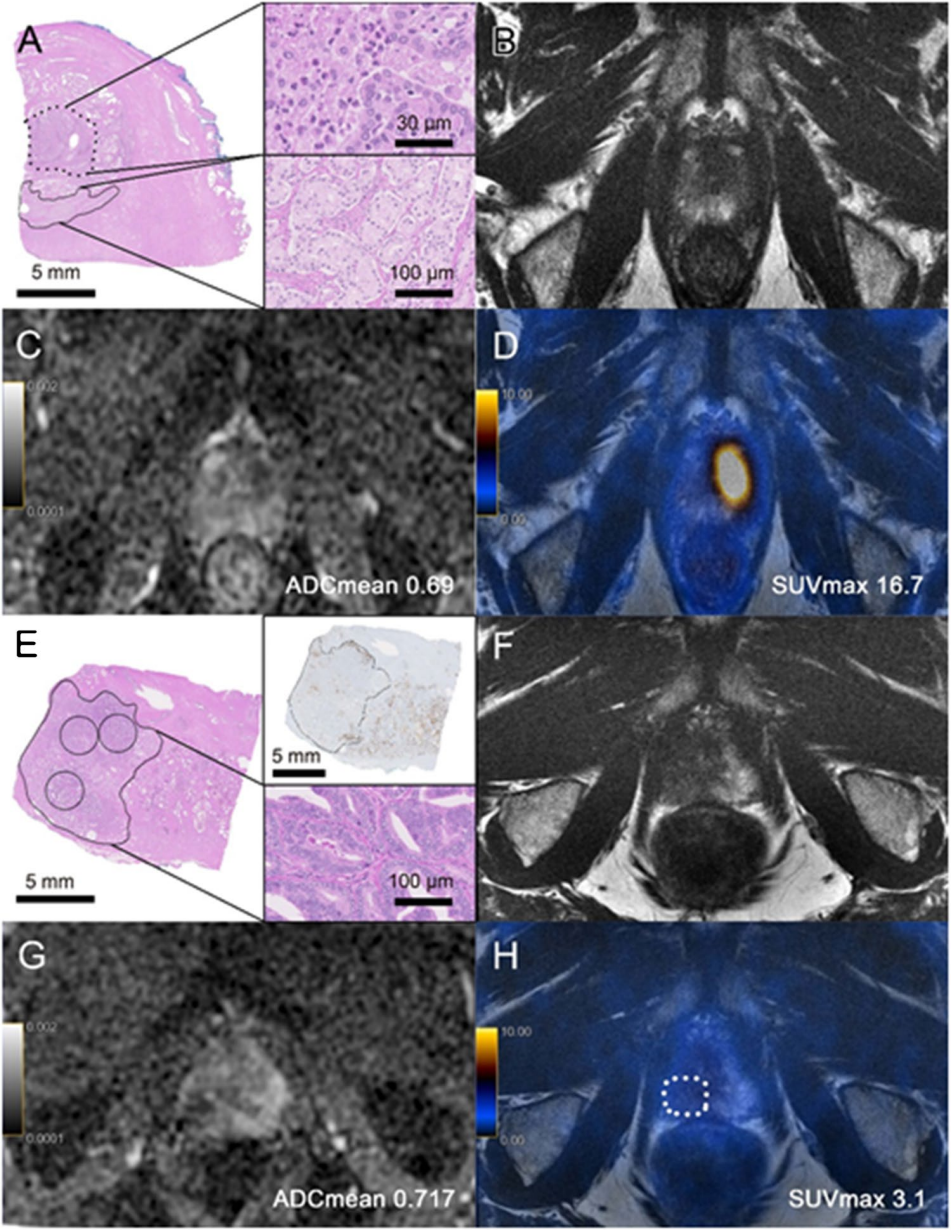
Two cases without correspondence between growth pattern and imaging parameters. **A**–**D** A 62-year-old patient with PSA of 12.7 ng/ml, GS = 4 + 3 (ISUP 3). **A** Histopathology slide showing an infiltrative growth pattern of the “dominant lesion’’ (continuous circle, detail on the right lower right side) on the anterior left part of the prostate apex with a maximum diameter of 8.3 mm; next to the carcinomatous lesion, acute inflammation (dotted circle, detail on the upper right side) can be found. **B** Corresponding axial T2-weighted MRI with a hypointense area. **C** On ADC map, the lesion had a low ADC_mean_ of 0.69. **D** On axial [^68^Ga]PSMA PET/MRI, the lesion showed high PSMA-uptake (SUV_max_ = 16.7). In direct correlation of imaging and histopathology, the acute inflammation was probably responsible for the low ADC value and high PSMA uptake. **E**–**H** A 60-year-old patient with PSA of 1.3 ng/ml, GS = 4 + 3 (ISUP 3). **E** Histopathology slide showing an expansive growth pattern of the large “dominant lesion’’ on the posterior right part of the prostate apex with a maximum diameter of 17.3 mm. **F** Corresponding axial T2-weighted MRI with a hypointense area. **G** On ADC map, the lesion had a low ADC_mean_ of 0.717. **H** On [^68^Ga]PSMA PET/MRI, the lesion showed low PSMA-uptake (SUV_max_ = 3.1)

We assessed the potential of SUV_max_ and ADC_mean_ to discriminate between EXP or INF growth, respectively. The AUC for SUV_max_ was 0.785 for EXP growth with an optimal cutoff at 13.0, yielding a sensitivity and specificity of 76.0% (19/25) and 83.7% (30/37), respectively (Supplemental Fig. 2A, Table 4). The AUC for ADC_mean_ was 0.850 for INF growth with an optimal cutoff at 0.955, yielding a sensitivity and specificity of 67.6% (25/37) and 100% (25/25), respectively (Supplemental Fig. 2B, Table 4). Not for all lesions this cutoff could predict the growth pattern; in Fig. 6, we show two cases without correspondence between growth pattern and imaging parameters.

**Table 4:** Contingency’s tables for PSMA SUV_max_ and ADC_mean_

	EXP growth	INF growth
PSMA SUV_max_ < 13.0	6/62	30/62
PSMA SUV_max_ > 13.0	19/62	7/62
ADC_mean_ < 0.955	25/62	12/62
ADC_mean_ > 0.955	0/62	25/62

*EXP*, expansive; *INF*, infiltrative; *PSMA*, prostate-specific membrane antigen; *SUV*, standardized uptake value; *max*, maximum; *ADC*, apparent diffusion coefficient

### Correlation of tumor size and imaging parameters

In the analysis of the impact of tumor size and ISUP on imaging parameters, we found a positive correlation between tumor size and SUV_max_ only for ISUP 3 or more, with a tendency for a stronger correlation for higher ISUP (Supplemental Fig. 3A). Overall, there was a moderate correlation for SUV_max_ and tumor size (*r* = 0.342, *p* = 0.006). For diffusion restriction, there was a strong negative correlation between tumor size and ADC_mean_ for all ISUP grades. The overall correlation for ADC_mean_ and tumor size was higher than for ADC_mean_ and ISUP: *r* =  − 0.523 (*p* < 0.001) vs *r* =  − 0.221 (*p* = 0.084), respectively (Supplemental Fig. 3B).

## Discussion

It is well-known that there is a strong inverse relationship between tumor cellularity and ADC values [29]. This was also confirmed in our small cohort, with a trend between ISUP and ADC_mean_. However, we found that the correlation between ADC values and ISUP grades is lost if the two growth patterns are investigated separately. This is probably due to the fact, that ADC was significantly higher in lesions with INF growth with ADC_mean_ 1.079 mm^2^/1000 s compared to EXP growth ADC_mean_ 0.777 mm^2^/1000 s, and that tumors with EXP growth had overall higher ISUP grades [20, 30].

Other groups already observed that dense tumors had significantly lower ADC values than sparse tumors; however, they considered only peripheral zone lesions and did not assess the correlation with GS: Rosenkrantz et al. found an association between histopathology growth pattern and detectability of tumor on mpMRI, with solid tumor growth (defined as at least 5 mm of a continuous tumor with loose stroma or minimal prevailing benign glands) associated with tumor detectability with an odds’ ratio of 37.6 [22]. Langer et al. also observed that tumors with a “spares” growth pattern (defined as more than 50% of the cross-sectional area with normal glandular tissue) had no reduction in ADC or T2 values compared to normal tissue [21]. Later, the same group used semiautomatic tissue segmentation to correlate histopathological features with MRI findings. They found that ADC and T2 values were negatively related to the percentage area of nuclei or cytoplasm but positively related to the percentage area of luminal space [23]. Recently, Shiradkar et al. used deep learning to explore the relationship between histopathology tissue composition and MRI fingerprinting within regions of PCa, prostatitis, and the normal peripheral zone. They confirmed that T2 and ADC values dropped with increasing ISUP and that the lumen ratio induced significantly higher values for T2 and ADC [31].

Classical solid tumor growth is the fundamental attribute to classify GS 5 disease [32]. A strong correlation between solid growth and ADC, therefore, agrees with the expected tumor behavior. In our cohort, 25 lesions showed an EXP growth, but among them, only eight had solid growth patterns, 12 lesions showed cribriform characteristics, six had an intraductal component, and five showed more than two morphology patterns (e.g., acinar, intraductal, and cribriform). Interestingly, in our cohort, ISUP grade 1–4 lesions with EXP growth and non-classical solid tumor components had low ADC values.

We, therefore, believe that growth pattern characteristics are not yet well understood and need further investigation. We defined an INF growth for entrapped benign glands within the carcinoma complexes, whereas an EXP growth showed pure carcinoma glands within an area of at least 3 circles 5 mm^2^ of each (radius 1.26 mm). With this simple and easy to implement definition, a robust, almost perfect, classification between EXP and INF growth was possible. The strong correlation between growth pattern and imaging parameters confirms that the morphology characteristics of histopathology have an impact on imaging parameters. Again, higher PSMA uptake was associated with EXP growth (SUV_max_ 19.2) compared to INF growth (SUV_max_ 9.3). This positive correlation has already been shown in a previous study [19].

The fact that cancer lesions are intermingled by benign glands in an INF growth was a potential explanation for lower SUV values regardless of the grading of the membranous or cytoplasmic immunohistochemistry staining [19]. According to Woythal et al., benign glands have lower PSMA expression and lower PSMA SUV_max_ than PCa. The same is true for diffusion restriction and ADC values on mpMRI; therefore, mixed tissue probably has higher ADC values [33]. Alternatively, a cumulative reduction of diffusion is typical of the aggressive disease being related to a dense growth pattern (Supplemental Fig. 4A-D). We showed in this study that there are PCa lesions with high ISUP but INF growth and high ADC values. The overall reduced density of those lesions might represent the substrate of the biologic principle able to explain false-negative ADC maps (Supplemental Fig. 4E-H).

Not all lesions with INF growth were difficult to localize on imaging, such as in one patient shown in Fig. 6A–D, with an ISUP 3 tumor and INF growth, the PSMA uptake was intense (SUV_max_ 16.7) and the diffusion restriction was very prominent with ADC_mean_ 0.69. This tumor had a maximal diameter of 8 mm, and there was an association with inflammatory changes that might have influenced the imaging characteristics. All EXP growth lesions had an ADC_min_ < 0.800 and were visible on ADC maps. Only two lesions with EXP growth had a SUV_max_ < 5 on PSMA PET; one is presented in Fig. 6E–H; both had large PSMA-negative tumor areas on immunohistochemistry.

Overall, we found that the correlation of ADC values was stronger between tumor size on histopathology than ISUP grade (*r* =  − 0.523, *p* < 0.001 vs *r* =  − 0.192, *p* = 0.135), while PSMA uptake was more dependent on ISUP grade than tumor size (*r* = 0.508, *p* < 0.001 vs *r* = 0.342, *p* = 0.006). These observations will need validation in larger external cohorts.

Limitations of this study are the retrospective design and the selection bias, given the inclusion of patients who underwent RPE and PSMA PET/MRI only. Furthermore, we lack the whole-mount preparation of histological slides, which would have simplified the correlation between imaging and histopathology. Also, 43/62 patients received a [^68^Ga]PSMA-11 mean dose of 129.8 ± 21.1 MBq (81–160), while 19/62 patients (biopsy guidance) received 85 MBq. Another limitation is that the proposed growth pattern is not an established feature on histopathology analysis for PCa. The exact definition of both patterns might still be subject to further adjustments (e.g., 3 × 5 mm^2^ is indeed the ideal size to define an EXP growth pattern). However, we observed that the proposed growth patterns are strongly associated with imaging parameters both on mpMRI and PSMA PET and could show a high reproducibility between pathologists.

This warrants further investigation, especially regarding the potential translation of growth pattern analysis to core biopsies. The awareness of the growth pattern based on biopsy might alter the interpretation of mpMRI, given the reduced reliability of ADC values.

## Conclusion

PCa with INF growth showed significantly lower SUV_max_ and higher ADC_mean_ values compared to EXP growth PCa and overall lower ISUP grades. The established negative correlation between ADC_mean_ and ISUP grade was not confirmed for separate analyses of both growth patterns.

## Supplementary information

Below is the link to the electronic supplementary material.

Supplementary file1 (DOCX 995 KB)

## Data Availability

Data are available for bona fide researchers who request it from the authors.
