# Supplementary material for: Infiltrative growth pattern of prostate cancer is associated with lower uptake on PSMA PET and reduced diffusion restriction on mpMRI
Source: Eur J Nucl Med Mol Imaging. 2022 Apr 18;49(11):3917–28. doi: 10.1007/s00259-022-05787-9 (PMC9399036; doi:10.1007/s00259-022-05787-9)
Supplement: Supplementary file 1 — Supplementary file1 (DOCX 995 KB) [file 259_2022_5787_MOESM1_ESM.docx]

**Supplemental Material**

**
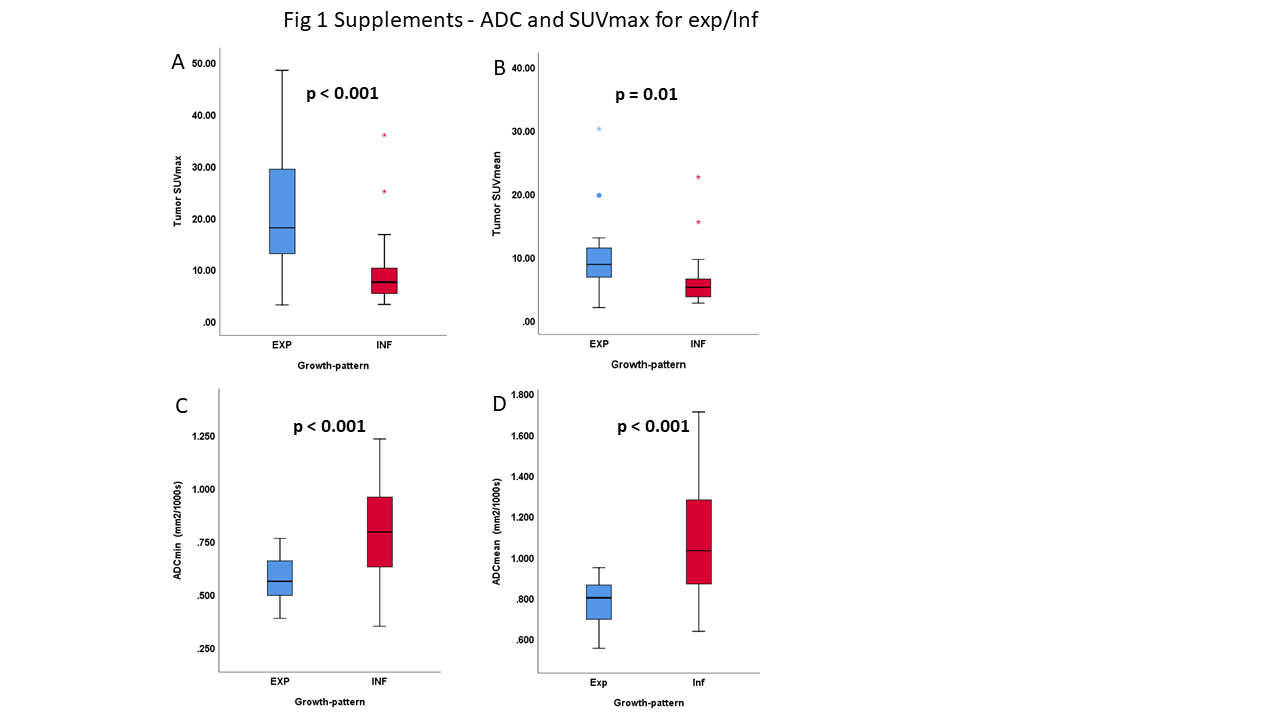
**

**Supplemental Figure 1.** Box plots for **A)** Tumor SUV_max_, **B)** SUV_mean_, **C)** ADC_min_, and **D)** ADC_mean_ values according to growth pattern.

**
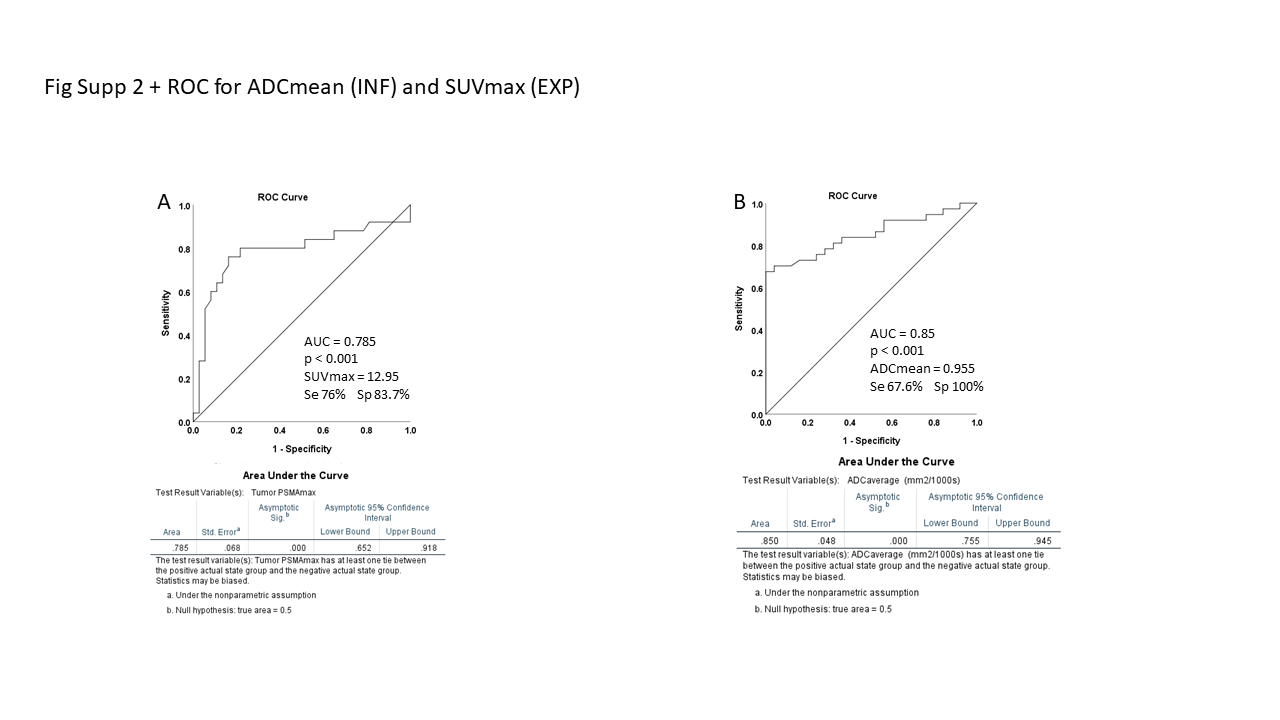

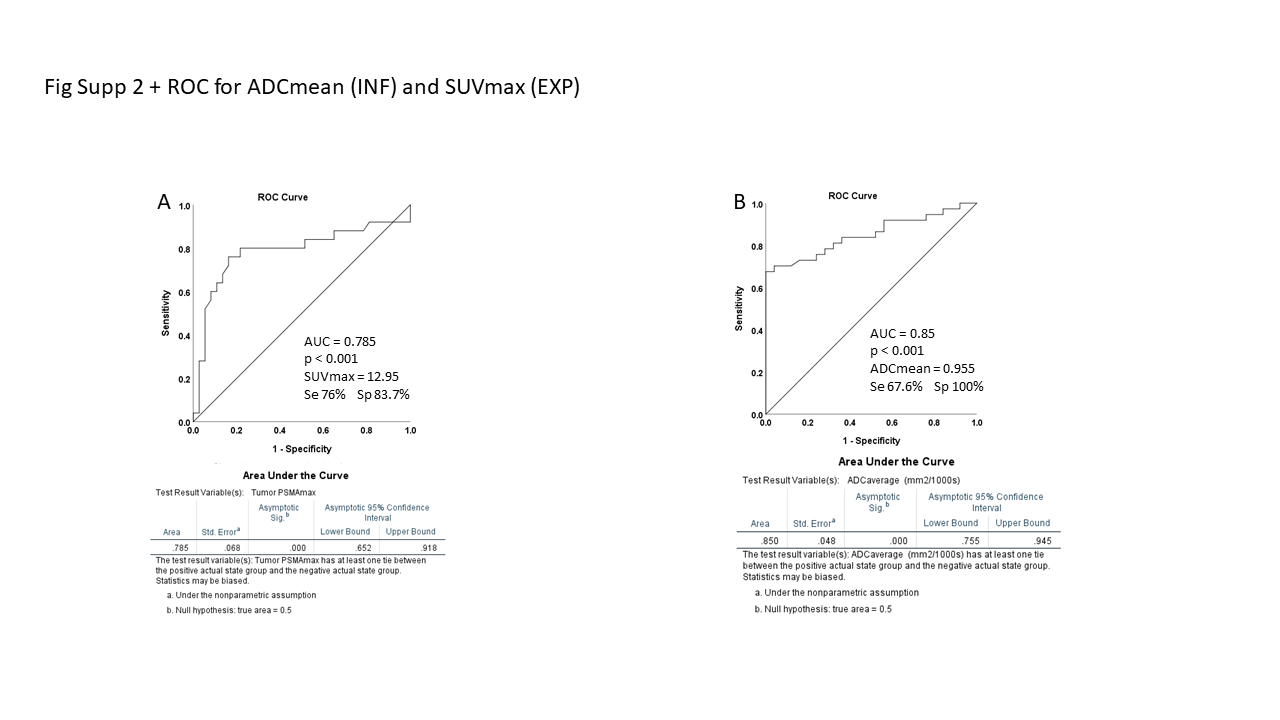
**

**Supplemental Figure 2. A)** Receiver operating characteristics (ROC) curve analysis for SUV_max_ prediction of EXP-growth. A SUV_max_ value of 13.0 reached a sensitivity of 76% and specificity of 83.7%. **B)** ROC curve analysis for ADC_mean_ prediction of INF-growth. An ADC_mean_ value of 0.955 reached a sensitivity of 67.6% and specificity of 100%.

**
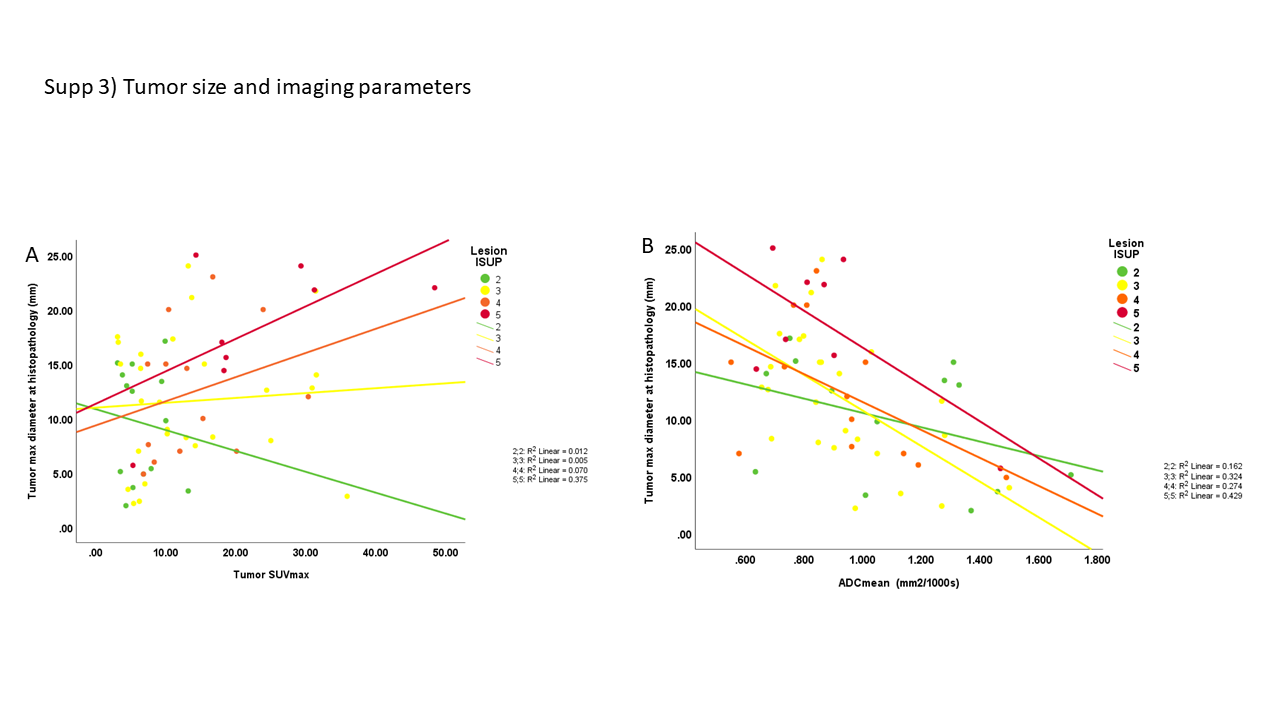
**

**Supplemental Figure 3. A)** Scatterplot for the maximum tumor diameter and SUV_max_ with color-coded ISUP grade to show the relationship between size, ISUP grade and SUV_max_. A linear fit for the different ISUP grades showed the following correlations: ISUP 2 10.86–0.19*x; ISUP 3 11 + 0.04*x; ISUP 4 9.37 + 0.22*x; ISUP 5 11.35+0.3*x. The same was done for ADC_mean_ **B**) inducing negative correlations for all ISUP grades: ISUP 2 15.34–5.32*x; ISUP 3 27.05–16.56*x; ISUP 4 23.76–12.24*x; ISUP 5 32.44–16.16*x. This illustrates that the correlation between tumor size and ADC_mean_ was independent from ISUP grades, while SUV_max_ had a positive correlation between size and PSMA uptake for higher-grade tumors (ISUP 4/5) only.


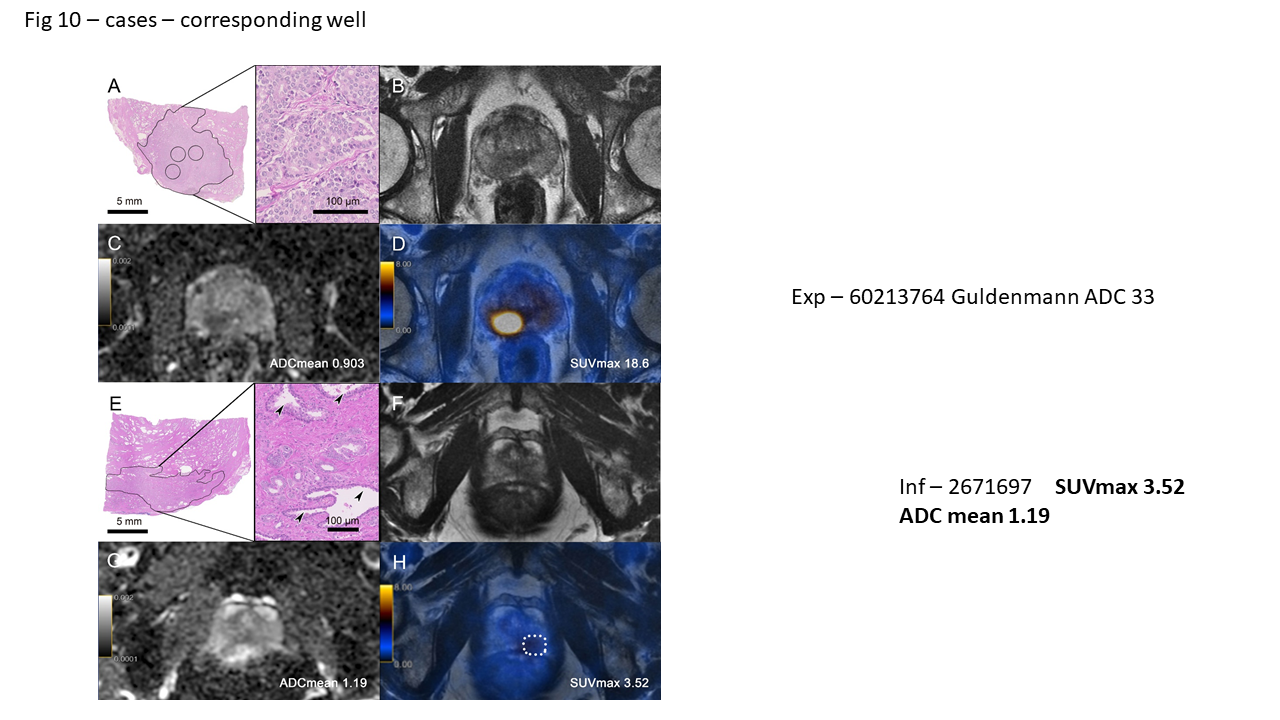


**Supplemental Figure 4.** Two cases with good correspondence between growth pattern and imaging parameters: **A-D)** 61-year-old patient with PSA of 9.8ng/ml, GS = 4+5 (ISUP 5). **A)** Histopathology slide showing an expansive growth-pattern of the “dominant lesion’’ on the posterior right peripheral zone of the prostate with 15.6mm maximum diameter, **B)** corresponding axial T2-weighted MRI with an extensive hypointense area; **C)** on ADC map the lesion had a low ADC_mean_ of 0.903 and on **D)** axial [^68^Ga]PSMA PET/MRI the lesion showed high PSMA-uptake (SUV_max_ = 18.6). **E-H**) 72-year-old patient with PSA of 7.85ng/ml, GS = 4+4 (ISUP 4). **E)** Histopathology slide showing an infiltrative growth-pattern of the ‘’dominant lesion’’ on the posterior left part of the prostate apex with 15mm maximum diameter; **F)** corresponding axial T2-weighted MRI without clear pathological area; **G)** on ADC map, the lesion had a high ADC_mean_ of 1.19 and on **H)** axial [^68^Ga]PSMA PET/MRI, the lesion showed low PSMA-uptake (SUV_max_ = 3.5).

**Supplement Table 1.** MRI sequence details

|  | Axial DWI EPI (Focus) (Pelvis) | Axial LAVA-FLEX WB (DIXON) | Axial T1w Whole ARC (Pelvis) | Axial T2w FRFSE-XL  (Pelvis) | Coronal T2w WB FRFSE-XL | Coronal T2w  FRFSE-XL  (Pelvis) | Axial DCE  (Lava Dyn)  (Pelvis) |
| --- | --- | --- | --- | --- | --- | --- | --- |
| Repetition time, TR (ms) | 4000 | 5.6 | 550 | 2600 | 6304 | 2900 | 6.361 |
|  |  |  |  |  |  |  |  |
| Echo time,  TE (ms) | 67.3 | 1.3-2.7 | 8.26 | 117 | 123 | 121 | 2.376 |
|  |  |  |  |  |  |  |  |
| Flip angle,  FA (degrees) | 90 | 12 | 111 | 125 | 111 | 125 | 30 |
|  |  |  |  |  |  |  |  |
| Acquisition matrix | 160 x 80 | 344 x 256 | 352 x 352 | 416 x 224 | 288 x 224 | 416 x 224 | 160 x 80 |
|  |  |  |  |  |  |  |  |
| Image size (voxels) | 256 x 256 | 512 x 512 | 512 x 512 | 512 x 512 | 512 x 512 | 512 x 512 | 288 x 192 |
|  |  |  |  |  |  |  |  |
|  |  |  |  |  |  |  |  |
| Slice thickness (mm) | 4 | 3 | 5 | 4 | 5 | 4 | 4 |
|  |  |  |  |  |  |  |  |
| Signal averages | 8 | 0.68 | 0.5 | 2 | 0.5 | 4 | 0.35 |
|  |  |  |  |  |  |  |  |
| b-values (s/mm2) and signal averages | 0 (6 av.)  400 (8 av.)  700(16 av.) |  |  |  |  |  |  |
|  |  |  |  |  |  |  |  |
| Diffusion direction | ‘All’ |  |  |  |  |  |  |
|  |  |  |  |  |  |  |  |
| Bandwidth (Hz/pixel) | 1953 | 166 | 1953 | 326 | 355 | 326 | 62.5 |
|  |  |  |  |  |  |  |  |
| Acquisition time (mm:ss) | 5:41 | 0:18 | 3:53 | 3:48 | 0:24 | 4:04 | 3:27 |
